# Supplementary material for: Clinical Efficacy and Safety of an Automatic Closed-Suction System in Mechanically Ventilated Patients with Pneumonia: A Multicenter, Prospective, Randomized, Non-Inferiority, Investigator-Initiated Trial
Source: Diagnostics (Basel). 2024 May 21;14(11):1068. doi: 10.3390/diagnostics14111068 (PMC11172224; doi:10.3390/diagnostics14111068)
Supplement: Supplementary file 1 [file diagnostics-14-01068-s001.zip › diagnostics-2972340-supplementary/Supplementary material S1.pdf]

Supplementary materials

Clinical efficacy and safety of an automatic closed-suction system in mechanically ventilated patients with pneumonia: A multicenter, prospective, randomized, non-inferiority, investigator-initiated trial

Dong-Hyun Joo, Hyo Chan Park, Joon Han Kim, Seo Hee Yang, Tae Hun Kim, Hyung-Jun Kim, Myung Jin Song, Sung Yoon Lim, Sung A Kim, Hee Won Bae, Yoon Hae Ahn, Si Mong Yoon, Jimyung Park, Hong Yeul Lee, Jinwoo Lee, Sang-Min Lee.

Affiliation and e-mail address of the corresponding author: Young-Jae Cho, M.D., M.P.H., Ph.D., Division of Pulmonary and Critical Care Medicine, Department of Internal Medicine, Seoul National University College of Medicine, Seoul National University Bundang Hospital, Republic of Korea; lungdrcho@snu.ac.kr

Supplementary material 1. Overview of the automatic suction device, Supplementary methods, Supplementary results.

Contents

S1. Overview of the automatic suction device

Figure S1. Overview of the automatic suction device, A-1000

S2. Inclusion and exclusion criteria

S3. Sample size estimation

Table S1. Evidence for sample size estimation

S4. Characteristics of the participants in the trial

Table S2. Detailed classification of study participants

S5. Baseline characteristics of the participants in the FA set

Table S3a. Baseline demographics of the participants in the FA set

Table S3b. Baseline characteristics of the participants in the FA set

Table S3c. Baseline vital signs of the participants in the FA set

Table S3d. Baseline electrocardiogram findings of the participants in the FA set

Table S3e. Baseline physical exam findings of the participants in the FA set

Table S3f. Baseline laboratory findings of the FA set

Table S3g. Baseline result of sputum culture of participants in the FA set

Table S3h. Baseline chest radiographic findings of participants in the FA set

Table S3i. Baseline tracheal secretions of the participants in the FA set

Table S3j. Baseline modified CPIS of the participants in the FA set

Table S3k. Baseline medical history of the participants in the FA set

Table S3l. Baseline present medical condition of the participants in the FA set

Table S3m. Baseline previous medication history of the participants in the FA set

Table S3n. Baseline concomitant medications of the participants in the FA set

S6. Efficacy outcome in the FA set

Table S4a. Primary efficacy outcome in the FA set

Table S4b. Secondary outcomes in the FA set

Table S4c. Number of suctions performed in addition to those specified in the protocol for 72 ± 3 h in the FA set

Table S4d. Total amount of secretions (cc) collected for 72 ± 3 h for participants in the FA set

Table S4e. Results of the device satisfaction survey after 72 ± 3 h in the FA set

S7. Safety outcome in the safety set

Table S5a. Summary of the adverse events that occurred during the study

Table S5b. Adverse events by organ system

Table S5c. Tracheal mucosal injury

Figure S2. Improvement in tracheal mucosal injury

S1. Overview of the automatic suction device

A-1000 is brand-new automatic closed suction system and it consists of a main body and consumable closed suction catheters. The principle of how it works is detailed in the attached online video file.

Figure S1. Overview of the automatic suction device, A-1000

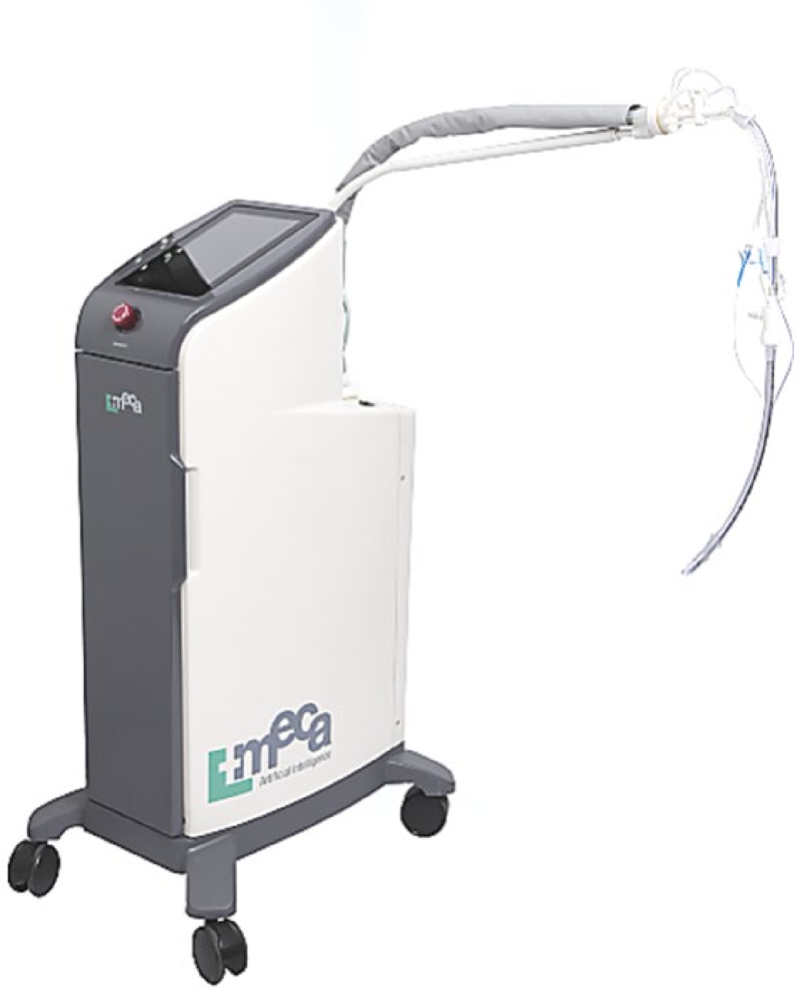

S2. Inclusion and exclusion criteria

S2.1 Inclusion criteria

1) Age ≥19 years

2) Diagnosis of pneumonia and requiring mechanical ventilation for >72 h

3) Voluntary decision to participate in the clinical trial and provide written informed consent to participate in the trial

S2.2 Exclusion criteria

1) Not being able to undergo bronchoscopy

2) Admission to the intensive care unit (ICU) for simple monitoring with expectations to be extubated early or with an APACHE II score of 30 points or higher when evaluated after 24 h of ICU admission or in a critical condition and expected to expire within 72 h

3) Requiring mechanical ventilation with higher than 80% FiO_2 or 30 cmH_2O in P_{plat} to maintain adequate oxygenation

4) Having severe immune deficiency (e.g., progressive hematologic malignancy, bone marrow graft failure, agranulocytosis, absolute neutrophil count <400/mm^3)

5) Having hemoptysis

6) Being pregnant or planning to become pregnant during the clinical trial

7) Underwent CPR within 5 days of screening

8) Judged difficult to apply the medical device by investigator considering the medical conditions

9) Currently participating in other clinical trial or had participated in other clinical trials within 90 days of the screening date

10) Judged to be inappropriate to participate in the trial because of ethical issue, or of possibility of influencing clinical trial results.

S2.3 The specific reason was written in the case report form

S3. Sample size estimation

S3.1 Estimated sample size

A total of 102 participants, with 51 patients in each group (including dropout rate of 20%)

S3.2 Evidence for sample size estimation

In this clinical trial, we planned to evaluate the non-inferiority of the automatic closed-suction system "A-1000" in mechanically ventilated patients with pneumonia compared with that of the conventional manual suction system in efficacy and safety.

The hypothesis to prove non-inferiority to conventional manual suction system was as followed.

$$H_o: \mu_t - \mu_c \geq \epsilon \; vs \; H_1: \mu_t - \mu_c < \epsilon$$

- μ_t : $mean\ chagne\ in\ modified\ CPIS\ in\ intervention\ (at\ 3\ days - baseline)$
- μ_c : $mean\ chagne\ in\ modified\ CPIS\ in\ control\ (at\ 3\ days - baseline)$
- ϵ: $Non - inferiority\ margin$

Modified CPIS was selected as a surrogate marker for evaluation. Using the following reference, the expected performance and non-inferiority margin for the intervention device and control method were estimated.

Supplementary Table S1. Evidence for sample size estimation

	**Day 0**			**Day 3**			**Diff CPIS**	
	n	mean	SD	N	mean	SD	Difference	95% CI
Cho JY et al.(2020)[1]	20	7.8	1.2	20	6	2.1	-1.8	(-2.89, -0.71)
Lee HW et al.(2015)[2]	93	6.89	1.35	93	5.6		-1.29	(-1.68, -0.90)
						**Mean**	**-1.55**	

We hypothesized that the change in CPIS for the conventional manual suction method would be similar to that for the automatic suction method and used the mean change and standard deviation in CI from

two different studies. A mean change in the CPIS of "−1.55" and standard deviation from larger study of "1.35" was selected. Using higher confidence interval of "−0.71" as acceptable limits and expected performance of mean "−1.55," non-inferiority margin was set as "-0.84 (−1.55 − (−0.71))".

The estimated sample size for the comparison of two groups with a non-inferiority margin of 0.84 and standard deviation of 1.35, under power of 80% and significance level of 2.5%; thus, a total of 102 participants (51 per group) were selected, considering a dropout rate of 20%.

$$n = \frac{2(z_\alpha + z_\beta)^2 \sigma^2}{(\epsilon - (\mu_c - \mu_t))^2}$$

S4. Characteristics of the participants enrolled in the trial

Table S2. Detailed classification of study participants

**Classification**	**Intervention**	**Control**	**Total**
**Screening**			57
Screening dropout			0 (0.00)
**Randomization**	28	29	57
Completion of the trial	23 (82.14)	24 (82.76)	47 (82.46)
Dropout during the trial	5 (17.86)	5 (17.24)	10 (17.54)
Inappropriate case due to comorbid conditions or adverse events determined by investigator	2 (40.00)	0 (0.00)	2 (20.00)
Consent withdrawal	0 (0.00)	1 (20.00)	1 (10.00)
Extubation within 3 days	0 (0.00)	4 (80.00)	4 (40.00)
Inappropriate case due to other cause determined by investigator	3 (60.00)	0 (0.00)	3 (30.00)

Note 1: N (%): number (percentage)

Note 2: Inappropriate cases due to other causes were excluded as follows:

1S-001: Changed to conventional manual suction after applying the intervention for 48 h in consideration of participant safety as determined by investigator

1S-013: Dropout due to transfer to other ward after diagnosis of COVID-19

2S-022: Dropout before application of the device due to difficulty in continuing the study because of a change in the participant's medical condition.

S5. Baseline characteristics of the participants in the FA set

Table S3a. Baseline demographics of the participants in the FA set

	**Intervention (N=26)**	**Control (N=28)**	**Total (N=54)**	**p-value**
**Age, years**				0.4935#
N	26	28	54	
Mean (SD)	66.08 (18.05)	71.46 (10.85)	68.87 (14.87)	
Median	70.50	72.50	71.50	
Min, Max	27.00, 87.00	36.00, 89.00	27.00, 89.00	
**Sex, N (%)**				0.7299&
Male	22 (84.62)	22 (78.57)	44 (81.48)	
Female	4 (15.38)	6 (21.43)	10 (18.52)	
**Height (cm)**				0.6137‡
N	26	28	54	
Mean (SD)	164.04 (7.62)	165.23 (9.43)	164.66 (8.54)	
Median	163.50	164.50	164.35	
Min, Max	148.00, 178.60	146.70, 187.60	146.70, 187.60	
**Weight (kg)**				0.5446#
N	26	28	54	
Mean (SD)	61.36 (12.64)	59.44 (15.61)	60.36 (14.16)	
Median	58.55	55.95	57.65	
Min, Max	43.90, 95.00	36.80, 101.40	36.80, 101.40	
**Childbearing period, N(%)**				0.4000&
**Yes**	1 (25.00)	0 (0.00)	1 (10.00)	
**Pregnancy test, N(%)**				
Yes	0 (0.00)	0 (0.00)	0 (0.00)	
No	1 (100.00)	0 (0.00)	1 (100.00)	
No	3 (75.00)	6 (100.00)	9 (90.00)	

	Intervention (N=26)	Control (N=28)	Total (N=54)	p-value
**Reason for non-childbearing period, N(%)**				
Before menarche	0 (0.00)	0 (0.00)	0 (0.00)	
Surgical sterilization	0 (0.00)	0 (0.00)	0 (0.00)	
Menopause	3 (100.00)	6 (100.00)	9 (100.00)	
Others	0 (0.00)	0 (0.00)	0 (0.00)	

‡: Independent two-sample *t*-test

#: Wilcoxon rank-sum test

&: Fisher's exact test

-: Not analyzed

Table S3b. Baseline characteristics of the participants in the FA set

	Intervention (N=26)	Control (N=28)	Total (N=54)	*p*-value
**Duration of pneumonia, day**				0.2071#
N	26	28	54	
Mean (SD)	6.58 (6.36)	10.50 (12.34)	8.61 (10.03)	
Median	5.00	6.00	5.00	
Min, Max	1.00, 29.00	1.00, 58.00	1.00, 58.00	
**APACHE II score**				0.3828‡
N	26	28	54	
Mean (SD)	19.73 (5.55)	20.89 (4.09)	20.33 (4.84)	
Median	20.00	20.00	20.00	
Min, Max	11.00, 29.00	11.00, 28.00	11.00, 29.00	
**Crackles, N (%)**				0.7426$
**Yes**	10 (38.46)	12 (42.86)	22 (40.74)	
**Location of crackles**				

	Intervention (N=26)	Control (N=28)	Total (N=54)	*p*-value
LULF	3 (30.00)	2 (16.67)	5 (22.73)	
LMLF	4 (40.00)	2 (16.67)	6 (27.27)	
LLLF	10 (100.00)	8 (66.67)	18 (81.82)	
RULF	3 (30.00)	2 (16.67)	5 (22.73)	
RMLF	3 (30.00)	3 (25.00)	6 (27.27)	
RLLF	7 (70.00)	7 (58.33)	14 (63.64)	
**No**	16 (61.54)	16 (57.14)	32 (59.26)	
**Endotracheal intubation, N (%)**				0.4815[&]
**Yes**	25 (96.15)	28 (100.00)	53 (98.15)	
**Inner diameter (mm)**				0.8345[#]
N	25	28	53	
Mean (SD)	7.62 (0.22)	7.61 (0.21)	7.61 (0.21)	
Median	7.50	7.50	7.50	
Min, Max	7.50, 8.00	7.50, 8.00	7.50, 8.00	
**Depth of intubation (mm)**				0.3771[#]
N	25	28	53	
Mean (SD)	229.60 (20.31)	230.00 (12.47)	229.81 (16.47)	
Median	230.00	230.00	230.00	
Min, Max	200.00, 300.00	200.00, 260.00	200.00, 300.00	
**No**	1 (3.85)	0 (0.00)	1 (1.85)	
**ICU admission, N(%)**				-
**Yes**	26 (100.00)	28 (100.00)	54 (100.00)	
**Duration in ICU, day**				0.6706[#]
N	26	28	54	
Mean (SD)	4.85 (6.58)	3.46 (2.70)	4.13 (4.96)	
Median	2.00	2.00	2.00	

	Intervention (N=26)	Control (N=28)	Total (N=54)	*p*-value
Min, Max	1.00, 32.00	1.00, 10.00	1.00, 32.00	
**No**	0 (0.00)	0 (0.00)	0 (0.00)	

Duration of pneumonia = [date of written informed consent − date of diagnosis of pneumonia] + 1

‡: Independent two-sample *t*-test

#: Wilcoxon rank-sum test

$: Chi-square test

&: Fisher's exact test

-: Not analyzed

Table S3c. Baseline vital signs of the participants in the FA set

	Intervention (N=26)	Control (N=28)	Total (N=54)	p-value
**Mean blood pressure (mmHg)**				0.4992#
N	26	28	54	
Mean (SD)	87.15 (13.73)	90.14 (15.55)	88.70 (14.64)	
Median	84.50	87.50	86.00	
Min, Max	68.00, 115.00	63.00, 142.00	63.00, 142.00	
**Heart rate (/min)**				0.4186‡
N	26	28	54	
Mean (SD)	92.42 (21.72)	97.32 (22.37)	94.96 (21.99)	
Median	89.50	95.00	92.50	
Min, Max	60.00, 153.00	62.00, 145.00	60.00, 153.00	
**Respiratory rate (/min)**				0.2291‡
N	26	28	54	
Mean (SD)	20.82 (4.97)	22.34 (4.19)	21.61 (4.61)	
Median	21.00	22.00	22.00	

Min, Max	11.00, 34.00	14.00, 30.80	11.00, 34.00	
**SpO_2, %**				0.4232#
N	26	28	54	
Mean (SD)	97.46 (2.30)	96.82 (2.72)	97.13 (2.53)	
Median	97.50	97.00	97.00	
Min, Max	92.00, 100.00	91.00, 100.00	91.00, 100.00	
**Body temperature (°C)**				0.4659‡
N	26	28	54	
Mean (SD)	36.94 (0.58)	36.82 (0.60)	36.88 (0.58)	
Median	37.05	36.85	36.90	
Min, Max	35.80, 38.00	35.90, 38.30	35.80, 38.30	
**ARDS, N(%)**				0.7607$
Yes	15 (57.69)	15 (53.57)	30 (55.56)	
No	11 (42.31)	13 (46.43)	24 (44.44)	

Note 1: Baseline vital signs at screening.

Note 2: Maximum value for each measurement was analyzed.

‡: Independent two-sample *t*-test

#: Wilcoxon rank-sum test

$: Chi-square test

Table S3d. Baseline electrocardiogram findings of the participants in the FA set

	Intervention (N=26)	Control (N=28)	Total (N=54)	p-value
**1-lead ECG, N (%)**				1.0000&
Normal/NCS	25 (100.00)	27 (96.43)	52 (98.11)	
CS	0 (0.00)	1 (3.57)	1 (1.89)	

NCS Not clinically significant, *CS* clinically significant

Note 1: ECG at screening.

Note 3: One in intervention group (2S-003): Did not undergo a screening ECG.

Note 4: One in control group (1S-019); Atrial fibrillation at screening. Labeled as CS

&: Fisher's exact test

Table S3e. Baseline physical examination findings of the participants in the FA set

	Intervention (N=26)	Control (N=28)	Total (N=54)	p-value
**Physical exam, N (%)**				-
Normal/NCS	26 (100.00)	28 (100.00)	54 (100.00)	
CS	0 (0.00)	0 (0.00)	0 (0.00)	

NCS Not clinically significant, *CS* clinically significant

Note 1: Physical exam (chest, cardiac, etc.) at screening.

Note 2: Recorded as CS if there was even one CS in any physical exam.

-: Not analyzed

Table S3f. Baseline laboratory findings of the participants in the FA set

	Intervention (N=26)	Control (N=28)	Total (N=54)	p-value
**Laboratory findings, N (%)**				-
Normal/NCS	26 (100.00)	28 (100.00)	54 (100.00)	
CS	0 (0.00)	0 (0.00)	0 (0.00)	

NCS Not clinically significant, *CS* clinically significant

Note 1: Routine CBC, chemistry, coagulation panel, and ABGA at screening.

Note 2: Recorded as CS if there was even one CS in any laboratory findings.

-: Not analyzed

Table S3g. Baseline result of sputum culture in the FA set

	Intervention (N=26)	Control (N=28)	Total (N=54)	p-value
**Sputum culture, N (%)**				0.6104$
Negative	21 (80.77)	21 (75.00)	42 (77.78)	
Positive	5 (19.23)	7 (25.00)	12 (22.22)	
Inadequate specimen	0 (0.00)	0 (0.00)	0 (0.00)	

Note 1: Detailed results as follows: 5 in the intervention group: 1S-001 (*Stenotrophomonas maltophilia*), 1S-008 (NCV2019), 1S-010 (NCV 2019), 2S-008 (*Klebsiella pneumoniae ssp pneumoniae*), 2S-009 (*Acinetobacter baumannii*); and 7 in the control group: 1S-004 (*K. pneumoniae*), 1S-017 (NCV 2019), 1S-023 (NCV 2019), 2S-001 (*Pneumocystis. carinii*), 2S-021 (*Enterobacter cloacae complex*), 2S-023 (*E. cloacae*), 2S-028 (*Corynebacterium striatum*)

$: Chi-square test

Table S3h. Baseline chest radiographic findings of the participants in the FA set

	Intervention (N=26)	Control (N=28)	Total (N=54)	p-value
**Distribution of infiltration, N (%)**				1.0000&
No	0 (0.00)	0 (0.00)	0 (0.00)	
Diffused	22 (84.62)	24 (85.71)	46 (85.19)	
Localized	4 (15.38)	4 (14.29)	8 (14.81)	

&: Fisher's exact test

Table S3i. Baseline tracheal secretions of the participants in the FA set

	Intervention (N=26)	Control (N=28)	Total (N=54)	p-value
**Tracheal secretion, N(%)**				0.2397&
Rare	10 (38.46)	7 (25.00)	17 (31.48)	
Abundant	15 (57.69)	16 (57.14)	31 (57.41)	
Abundant + Purulent	1 (3.85)	5 (17.86)	6 (11.11)	

&: Fisher's exact test

Table S3j. Baseline modified CPIS of the participants in the FA set

	Intervention (N=26)	Control (N=28)	Total (N=54)	p-value
**Modified CPIS (0–12)**				0.9509#
N	26	28	54	
Mean (SD)	3.65 (1.85)	3.71 (1.86)	3.69 (1.84)	
Median	3.00	4.00	3.00	
Min, Max	1.00, 7.00	1.00, 7.00	1.00, 7.00	

#: Wilcoxon rank-sum test

Table S3k. Baseline medical history of the participants in the FA set

**System Organ Class** / **Preferred Terms**	**Intervention (N=26)**		**Control (N=28)**		**Total (N=54)**	
	**N (%)**	**E**	**N (%)**	**E**	**N (%)**	**E**
**Total**	**26 (100.00)**	**139**	**28 (100.00)**	**137**	**54 (100.00)**	**276**
p-value	-					
**Respiratory, thoracic and mediastinal disorders**	**20 (76.92)**	**28**	**16 (57.14)**	**26**	**36 (66.67)**	**54**
Acute respiratory distress syndrome	15 (57.69)	15	15 (53.57)	15	30 (55.56)	30

System Organ Class<br>Preferred Terms	Intervention (N=26)		Control (N=28)		Total (N=54)	
	N (%)	E	N (%)	E	N (%)	E
Idiopathic pulmonary fibrosis	2 (7.69)	2	3 (10.71)	3	5 (9.26)	5
Interstitial lung disease	3 (11.54)	3	2 (7.14)	2	5 (9.26)	5
Bronchiectasis	2 (7.69)	2	1 (3.57)	1	3 (5.56)	3
Chronic obstructive pulmonary disease	1 (3.85)	1	1 (3.57)	1	2 (3.70)	2
Emphysema	1 (3.85)	1	1 (3.57)	1	2 (3.70)	2
Respiratory failure	1 (3.85)	1	1 (3.57)	1	2 (3.70)	2
Asthma	1 (3.85)	1	0 (0.00)	0	1 (1.85)	1
Lung infiltration	0 (0.00)	0	1 (3.57)	1	1 (1.85)	1
Obstructive sleep apnea syndrome	1 (3.85)	1	0 (0.00)	0	1 (1.85)	1
Pleural effusion	1 (3.85)	1	0 (0.00)	0	1 (1.85)	1
Pneumothorax	0 (0.00)	0	1 (3.57)	1	1 (1.85)	1
**Vascular disorders**	**15 (57.69)**	**17**	**18 (64.29)**	**20**	**33 (61.11)**	**37**
Hypertension	15 (57.69)	15	18 (64.29)	18	33 (61.11)	33
Aortic aneurysm	1 (3.85)	1	0 (0.00)	0	1 (1.85)	1
Aortic stenosis	1 (3.85)	1	0 (0.00)	0	1 (1.85)	1
Arteriosclerosis	0 (0.00)	0	1 (3.57)	1	1 (1.85)	1
Deep vein thrombosis	0 (0.00)	0	1 (3.57)	1	1 (1.85)	1
**Metabolism and nutrition disorders**	**11 (42.31)**	**12**	**13 (46.43)**	**15**	**24 (44.44)**	**27**
Diabetes mellitus	9 (34.62)	9	11 (39.29)	11	20 (37.04)	20
Dyslipidemia	2 (7.69)	2	1 (3.57)	1	3 (5.56)	3
Hyperlipidemia	1 (3.85)	1	2 (7.14)	2	3 (5.56)	3
Hyponatremia	0 (0.00)	0	1 (3.57)	1	1 (1.85)	1
**Infections and infestations**	**10 (38.46)**	**16**	**9 (32.14)**	**13**	**19 (35.19)**	**29**
COVID-19	4 (15.38)	4	3 (10.71)	3	7 (12.96)	7
Atypical mycobacterial infection	1 (3.85)	1	1 (3.57)	1	2 (3.70)	2
Chronic hepatitis B	1 (3.85)	1	1 (3.57)	1	2 (3.70)	2
Kidney infection	1 (3.85)	1	1 (3.57)	1	2 (3.70)	2

System Organ Class<br>Preferred Terms	Intervention (N=26)		Control (N=28)		Total (N=54)	
	N (%)	E	N (%)	E	N (%)	E
Oral candidiasis	1 (3.85)	1	1 (3.57)	1	2 (3.70)	2
Urinary tract infection	1 (3.85)	1	1 (3.57)	1	2 (3.70)	2
Aspergilloma	1 (3.85)	1	0 (0.00)	0	1 (1.85)	1
Bacteremia	1 (3.85)	1	0 (0.00)	0	1 (1.85)	1
Bronchopulmonary aspergillosis	0 (0.00)	0	1 (3.57)	1	1 (1.85)	1
Hepatitis B	0 (0.00)	0	1 (3.57)	1	1 (1.85)	1
Herpes simplex	0 (0.00)	0	1 (3.57)	1	1 (1.85)	1
Herpes zoster	1 (3.85)	1	0 (0.00)	0	1 (1.85)	1
Pneumonia aspiration	0 (0.00)	0	1 (3.57)	1	1 (1.85)	1
Pyelonephritis, acute	1 (3.85)	1	0 (0.00)	0	1 (1.85)	1
Rhinitis	1 (3.85)	1	0 (0.00)	0	1 (1.85)	1
Septic shock	1 (3.85)	1	0 (0.00)	0	1 (1.85)	1
Skin bacterial infection	1 (3.85)	1	0 (0.00)	0	1 (1.85)	1
Tuberculosis	0 (0.00)	0	1 (3.57)	1	1 (1.85)	1
**Neoplasms: benign, malignant and unspecified (including cysts and polyps)**	**8 (30.77)**	**10**	**11 (39.29)**	**12**	**19 (35.19)**	**22**
Lung neoplasm malignant	2 (7.69)	2	6 (21.43)	6	8 (14.81)	8
Colon cancer	1 (3.85)	1	1 (3.57)	1	2 (3.70)	2
Acute lymphocytic leukemia	0 (0.00)	0	1 (3.57)	1	1 (1.85)	1
Acute promyelocytic leukemia	0 (0.00)	0	1 (3.57)	1	1 (1.85)	1
Chronic myeloid leukemia	1 (3.85)	1	0 (0.00)	0	1 (1.85)	1
Hepatocellular carcinoma	0 (0.00)	0	1 (3.57)	1	1 (1.85)	1
Leukemia	1 (3.85)	1	0 (0.00)	0	1 (1.85)	1
Lung cancer metastatic	1 (3.85)	1	0 (0.00)	0	1 (1.85)	1
Plasma cell myeloma	0 (0.00)	0	1 (3.57)	1	1 (1.85)	1
Plasmablastic lymphoma	1 (3.85)	1	0 (0.00)	0	1 (1.85)	1
Prostate cancer	1 (3.85)	1	0 (0.00)	0	1 (1.85)	1

System Organ Class<br>Preferred Terms	Intervention (N=26)		Control (N=28)		Total (N=54)	
	N (%)	E	N (%)	E	N (%)	E
Renal cell carcinoma	1 (3.85)	1	0 (0.00)	0	1 (1.85)	1
Thymoma malignant	0 (0.00)	0	1 (3.57)	1	1 (1.85)	1
Thyroid cancer	1 (3.85)	1	0 (0.00)	0	1 (1.85)	1
**Cardiac disorders**	**10 (38.46)**	**15**	**7 (25.00)**	**10**	**17 (31.48)**	**25**
Atrial fibrillation	3 (11.54)	3	3 (10.71)	3	6 (11.11)	6
Sinus tachycardia	3 (11.54)	3	1 (3.57)	1	4 (7.41)	4
Angina pectoris	1 (3.85)	1	1 (3.57)	1	2 (3.70)	2
Angina unstable	0 (0.00)	0	2 (7.14)	2	2 (3.70)	2
Coronary artery disease	2 (7.69)	2	0 (0.00)	0	2 (3.70)	2
Sinus bradycardia	0 (0.00)	0	2 (7.14)	2	2 (3.70)	2
Atrial flutter	1 (3.85)	1	0 (0.00)	0	1 (1.85)	1
Cardiac failure acute	1 (3.85)	1	0 (0.00)	0	1 (1.85)	1
Cardiac failure congestive	1 (3.85)	1	0 (0.00)	0	1 (1.85)	1
Mitral valve incompetence	1 (3.85)	1	0 (0.00)	0	1 (1.85)	1
Stress cardiomyopathy	1 (3.85)	1	0 (0.00)	0	1 (1.85)	1
Supraventricular tachycardia	0 (0.00)	0	1 (3.57)	1	1 (1.85)	1
Tricuspid valve incompetence	1 (3.85)	1	0 (0.00)	0	1 (1.85)	1
**Nervous system disorders**	**5 (19.23)**	**5**	**10 (35.71)**	**10**	**15 (27.78)**	**15**
Dementia	1 (3.85)	1	3 (10.71)	3	4 (7.41)	4
Parkinson's disease	1 (3.85)	1	2 (7.14)	2	3 (5.56)	3
Parkinsonism	1 (3.85)	1	1 (3.57)	1	2 (3.70)	2
Amyotrophic lateral sclerosis	0 (0.00)	0	1 (3.57)	1	1 (1.85)	1
Cerebrovascular accident	0 (0.00)	0	1 (3.57)	1	1 (1.85)	1
Embolic cerebral infarction	0 (0.00)	0	1 (3.57)	1	1 (1.85)	1
Epilepsy	1 (3.85)	1	0 (0.00)	0	1 (1.85)	1
Lacunar infarction	1 (3.85)	1	0 (0.00)	0	1 (1.85)	1
Myasthenia gravis	0 (0.00)	0	1 (3.57)	1	1 (1.85)	1

System Organ Class<br>Preferred Terms	Intervention (N=26)		Control (N=28)		Total (N=54)	
	N (%)	E	N (%)	E	N (%)	E
**Musculoskeletal and connective tissue disorders**	**5 (19.23)**	**7**	**5 (17.86)**	**7**	**10 (18.52)**	**14**
Osteoporosis	1 (3.85)	1	2 (7.14)	2	3 (5.56)	3
Rheumatoid arthritis	1 (3.85)	1	1 (3.57)	1	2 (3.70)	2
Spinal stenosis	0 (0.00)	0	2 (7.14)	2	2 (3.70)	2
Connective tissue disorder	1 (3.85)	1	0 (0.00)	0	1 (1.85)	1
Osteoarthritis	1 (3.85)	1	0 (0.00)	0	1 (1.85)	1
Osteonecrosis	1 (3.85)	1	0 (0.00)	0	1 (1.85)	1
Osteopenia	1 (3.85)	1	0 (0.00)	0	1 (1.85)	1
Polymyalgia rheumatica	0 (0.00)	0	1 (3.57)	1	1 (1.85)	1
Rotator cuff syndrome	0 (0.00)	0	1 (3.57)	1	1 (1.85)	1
Systemic lupus erythematosus	1 (3.85)	1	0 (0.00)	0	1 (1.85)	1
**Hepatobiliary disorders**	**5 (19.23)**	**5**	**4 (14.29)**	**4**	**9 (16.67)**	**9**
Hepatic cirrhosis	1 (3.85)	1	2 (7.14)	2	3 (5.56)	3
Autoimmune hepatitis	0 (0.00)	0	1 (3.57)	1	1 (1.85)	1
Bile duct stone	1 (3.85)	1	0 (0.00)	0	1 (1.85)	1
Cholecystitis acute	0 (0.00)	0	1 (3.57)	1	1 (1.85)	1
Chronic hepatitis	1 (3.85)	1	0 (0.00)	0	1 (1.85)	1
Hepatic steatosis	1 (3.85)	1	0 (0.00)	0	1 (1.85)	1
Liver disorder	1 (3.85)	1	0 (0.00)	0	1 (1.85)	1
**Renal and urinary disorders**	**4 (15.38)**	**4**	**3 (10.71)**	**4**	**7 (12.96)**	**8**
Acute kidney injury	1 (3.85)	1	2 (7.14)	2	3 (5.56)	3
Chronic kidney disease	1 (3.85)	1	1 (3.57)	1	2 (3.70)	2
Bladder dysfunction	0 (0.00)	0	1 (3.57)	1	1 (1.85)	1
Dysuria	1 (3.85)	1	0 (0.00)	0	1 (1.85)	1
Hydronephrosis	1 (3.85)	1	0 (0.00)	0	1 (1.85)	1
**Blood and lymphatic system disorders**	**2 (7.69)**	**2**	**4 (14.29)**	**4**	**6 (11.11)**	**6**

System Organ Class<br>Preferred Terms	Intervention (N=26)		Control (N=28)		Total (N=54)	
	N (%)	E	N (%)	E	N (%)	E
Anemia	1 (3.85)	1	3 (10.71)	3	4 (7.41)	4
Aplastic anemia	0 (0.00)	0	1 (3.57)	1	1 (1.85)	1
Thrombocytopenia	1 (3.85)	1	0 (0.00)	0	1 (1.85)	1
**Psychiatric disorders**	**3 (11.54)**	**3**	**2 (7.14)**	**2**	**5 (9.26)**	**5**
Depression	2 (7.69)	2	1 (3.57)	1	3 (5.56)	3
Depressed mood	1 (3.85)	1	0 (0.00)	0	1 (1.85)	1
Insomnia	0 (0.00)	0	1 (3.57)	1	1 (1.85)	1
**Gastrointestinal disorders**	**4 (15.38)**	**4**	**0 (0.00)**	**0**	**4 (7.41)**	**4**
Gastroesophageal reflux disease	2 (7.69)	2	0 (0.00)	0	2 (3.70)	2
Diarrhea	1 (3.85)	1	0 (0.00)	0	1 (1.85)	1
Superior mesenteric artery dissection	1 (3.85)	1	0 (0.00)	0	1 (1.85)	1
**Surgical and medical procedures**	**2 (7.69)**	**3**	**2 (7.14)**	**2**	**4 (7.41)**	**5**
Cord blood transplant therapy	0 (0.00)	0	1 (3.57)	1	1 (1.85)	1
Mastoidectomy	0 (0.00)	0	1 (3.57)	1	1 (1.85)	1
Pleurodesis	1 (3.85)	1	0 (0.00)	0	1 (1.85)	1
Thoracic cavity drainage	1 (3.85)	1	0 (0.00)	0	1 (1.85)	1
Tracheostomy	1 (3.85)	1	0 (0.00)	0	1 (1.85)	1
**Injury, poisoning, and procedural complications**	**1 (3.85)**	**1**	**2 (7.14)**	**2**	**3 (5.56)**	**3**
Radiation pneumonitis	0 (0.00)	0	1 (3.57)	1	1 (1.85)	1
Spinal cord injury	1 (3.85)	1	0 (0.00)	0	1 (1.85)	1
Thermal burn	0 (0.00)	0	1 (3.57)	1	1 (1.85)	1
**Reproductive system and breast disorders**	**1 (3.85)**	**1**	**2 (7.14)**	**2**	**3 (5.56)**	**3**
Benign prostatic hyperplasia	1 (3.85)	1	2 (7.14)	2	3 (5.56)	3
**Endocrine disorders**	**1 (3.85)**	**1**	**1 (3.57)**	**1**	**2 (3.70)**	**2**
Hypothyroidism	1 (3.85)	1	1 (3.57)	1	2 (3.70)	2
**Investigations**	**1 (3.85)**	**1**	**1 (3.57)**	**1**	**2 (3.70)**	**2**

System Organ Class<br>Preferred Terms	Intervention (N=26)		Control (N=28)		Total (N=54)	
	N (%)	E	N (%)	E	N (%)	E
Aspiration pleural cavity	0 (0.00)	0	1 (3.57)	1	1 (1.85)	1
Endoscopic retrograde cholangiopancreatography	1 (3.85)	1	0 (0.00)	0	1 (1.85)	1
**Skin and subcutaneous tissue disorders**	**1 (3.85)**	**1**	**1 (3.57)**	**1**	**2 (3.70)**	**2**
Dermatomyositis	1 (3.85)	1	0 (0.00)	0	1 (1.85)	1
Skin necrosis	0 (0.00)	0	1 (3.57)	1	1 (1.85)	1
**Ear and labyrinth disorders**	**1 (3.85)**	**1**	**0 (0.00)**	**0**	**1 (1.85)**	**1**
Hypoacusis	1 (3.85)	1	0 (0.00)	0	1 (1.85)	1
**Eye disorders**	**0 (0.00)**	**0**	**1 (3.57)**	**1**	**1 (1.85)**	**1**
Macular degeneration	0 (0.00)	0	1 (3.57)	1	1 (1.85)	1
**General disorders and administration site conditions**	**1 (3.85)**	**1**	**0 (0.00)**	**0**	**1 (1.85)**	**1**
Pyrexia	1 (3.85)	1	0 (0.00)	0	1 (1.85)	1
**Immune system disorders**	**1 (3.85)**	**1**	**0 (0.00)**	**0**	**1 (1.85)**	**1**
Graft versus host disease	1 (3.85)	1	0 (0.00)	0	1 (1.85)	1

Note 1: Duplication permitted.

Note 2: Coding by System Organ Class (SOC) and Preferred Term (PT) of MedDRA (version 26.0)

-: Not analyzed

Table S3l. Baseline medical condition of the participants in the FA set

System Organ Class<br>Preferred Terms	Intervention (N=26)		Control (N=28)		Total (N=54)	
	N (%)	E	N (%)	E	N (%)	E
**Total**	**26 (100.00)**	**128**	**28 (100.00)**	**130**	**54 (100.00)**	**258**
p-value	-					
**Respiratory, thoracic, and mediastinal disorders**	**20 (76.92)**	**28**	**16 (57.14)**	**26**	**36 (66.67)**	**54**
Acute respiratory distress syndrome	15 (57.69)	15	15 (53.57)	15	30 (55.56)	30
Idiopathic pulmonary fibrosis	2 (7.69)	2	3 (10.71)	3	5 (9.26)	5
Interstitial lung disease	3 (11.54)	3	2 (7.14)	2	5 (9.26)	5
Bronchiectasis	2 (7.69)	2	1 (3.57)	1	3 (5.56)	3
Chronic obstructive pulmonary disease	1 (3.85)	1	1 (3.57)	1	2 (3.70)	2
Emphysema	1 (3.85)	1	1 (3.57)	1	2 (3.70)	2
Respiratory failure	1 (3.85)	1	1 (3.57)	1	2 (3.70)	2
Asthma	1 (3.85)	1	0 (0.00)	0	1 (1.85)	1
Lung infiltration	0 (0.00)	0	1 (3.57)	1	1 (1.85)	1
Obstructive sleep apnea syndrome	1 (3.85)	1	0 (0.00)	0	1 (1.85)	1
Pleural effusion	1 (3.85)	1	0 (0.00)	0	1 (1.85)	1
Pneumothorax	0 (0.00)	0	1 (3.57)	1	1 (1.85)	1
**Vascular disorders**	**15 (57.69)**	**17**	**18 (64.29)**	**20**	**33 (61.11)**	**37**
Hypertension	15 (57.69)	15	18 (64.29)	18	33 (61.11)	33
Aortic aneurysm	1 (3.85)	1	0 (0.00)	0	1 (1.85)	1
Aortic stenosis	1 (3.85)	1	0 (0.00)	0	1 (1.85)	1
Arteriosclerosis	0 (0.00)	0	1 (3.57)	1	1 (1.85)	1
Deep vein thrombosis	0 (0.00)	0	1 (3.57)	1	1 (1.85)	1
**Metabolism and nutrition disorders**	**11 (42.31)**	**12**	**13 (46.43)**	**15**	**24 (44.44)**	**27**
Diabetes mellitus	9 (34.62)	9	11 (39.29)	11	20 (37.04)	20
Dyslipidemia	2 (7.69)	2	1 (3.57)	1	3 (5.56)	3

System Organ Class<br>Preferred Terms	Intervention (N=26)		Control (N=28)		Total (N=54)	
	N (%)	E	N (%)	E	N (%)	E
Hyperlipidemia	1 (3.85)	1	2 (7.14)	2	3 (5.56)	3
Hyponatremia	0 (0.00)	0	1 (3.57)	1	1 (1.85)	1
**Neoplasms: benign, malignant, and unspecified (including cysts and polyps)**	**8 (30.77)**	**10**	**11 (39.29)**	**12**	**19 (35.19)**	**22**
Lung neoplasm malignant	2 (7.69)	2	6 (21.43)	6	8 (14.81)	8
Colon cancer	1 (3.85)	1	1 (3.57)	1	2 (3.70)	2
Acute lymphocytic leukemia	0 (0.00)	0	1 (3.57)	1	1 (1.85)	1
Acute promyelocytic leukemia	0 (0.00)	0	1 (3.57)	1	1 (1.85)	1
Chronic myeloid leukemia	1 (3.85)	1	0 (0.00)	0	1 (1.85)	1
Hepatocellular carcinoma	0 (0.00)	0	1 (3.57)	1	1 (1.85)	1
Leukemia	1 (3.85)	1	0 (0.00)	0	1 (1.85)	1
Lung cancer metastatic	1 (3.85)	1	0 (0.00)	0	1 (1.85)	1
Plasma cell myeloma	0 (0.00)	0	1 (3.57)	1	1 (1.85)	1
Plasmablastic lymphoma	1 (3.85)	1	0 (0.00)	0	1 (1.85)	1
Prostate cancer	1 (3.85)	1	0 (0.00)	0	1 (1.85)	1
Renal cell carcinoma	1 (3.85)	1	0 (0.00)	0	1 (1.85)	1
Thymoma malignant	0 (0.00)	0	1 (3.57)	1	1 (1.85)	1
Thyroid cancer	1 (3.85)	1	0 (0.00)	0	1 (1.85)	1
**Cardiac disorders**	**10 (38.46)**	**14**	**7 (25.00)**	**10**	**17 (31.48)**	**24**
Atrial fibrillation	2 (7.69)	2	3 (10.71)	3	5 (9.26)	5
Sinus tachycardia	3 (11.54)	3	1 (3.57)	1	4 (7.41)	4
Angina pectoris	1 (3.85)	1	1 (3.57)	1	2 (3.70)	2
Angina unstable	0 (0.00)	0	2 (7.14)	2	2 (3.70)	2
Coronary artery disease	2 (7.69)	2	0 (0.00)	0	2 (3.70)	2
Sinus bradycardia	0 (0.00)	0	2 (7.14)	2	2 (3.70)	2
Atrial flutter	1 (3.85)	1	0 (0.00)	0	1 (1.85)	1
Cardiac failure acute	1 (3.85)	1	0 (0.00)	0	1 (1.85)	1

System Organ Class<br>Preferred Terms	Intervention (N=26)		Control (N=28)		Total (N=54)	
	N (%)	E	N (%)	E	N (%)	E
Cardiac failure congestive	1 (3.85)	1	0 (0.00)	0	1 (1.85)	1
Mitral valve incompetence	1 (3.85)	1	0 (0.00)	0	1 (1.85)	1
Stress cardiomyopathy	1 (3.85)	1	0 (0.00)	0	1 (1.85)	1
Supraventricular tachycardia	0 (0.00)	0	1 (3.57)	1	1 (1.85)	1
Tricuspid valve incompetence	1 (3.85)	1	0 (0.00)	0	1 (1.85)	1
**Infections and infestations**	**9 (34.62)**	**12**	**7 (25.00)**	**8**	**16 (29.63)**	**20**
Atypical mycobacterial infection	1 (3.85)	1	1 (3.57)	1	2 (3.70)	2
COVID-19	1 (3.85)	1	1 (3.57)	1	2 (3.70)	2
Chronic hepatitis B	1 (3.85)	1	1 (3.57)	1	2 (3.70)	2
Kidney infection	1 (3.85)	1	1 (3.57)	1	2 (3.70)	2
Urinary tract infection	1 (3.85)	1	1 (3.57)	1	2 (3.70)	2
Aspergilloma	1 (3.85)	1	0 (0.00)	0	1 (1.85)	1
Bacteremia	1 (3.85)	1	0 (0.00)	0	1 (1.85)	1
Bronchopulmonary aspergillosis	0 (0.00)	0	1 (3.57)	1	1 (1.85)	1
Hepatitis B	0 (0.00)	0	1 (3.57)	1	1 (1.85)	1
Herpes zoster	1 (3.85)	1	0 (0.00)	0	1 (1.85)	1
Oral candidiasis	1 (3.85)	1	0 (0.00)	0	1 (1.85)	1
Pneumonia aspiration	0 (0.00)	0	1 (3.57)	1	1 (1.85)	1
Pyelonephritis, acute	1 (3.85)	1	0 (0.00)	0	1 (1.85)	1
Rhinitis	1 (3.85)	1	0 (0.00)	0	1 (1.85)	1
Septic shock	1 (3.85)	1	0 (0.00)	0	1 (1.85)	1
**Nervous system disorders**	**5 (19.23)**	**5**	**10 (35.71)**	**10**	**15 (27.78)**	**15**
Dementia	1 (3.85)	1	3 (10.71)	3	4 (7.41)	4
Parkinson's disease	1 (3.85)	1	2 (7.14)	2	3 (5.56)	3
Parkinsonism	1 (3.85)	1	1 (3.57)	1	2 (3.70)	2
Amyotrophic lateral sclerosis	0 (0.00)	0	1 (3.57)	1	1 (1.85)	1
Cerebrovascular accident	0 (0.00)	0	1 (3.57)	1	1 (1.85)	1

System Organ Class<br>Preferred Terms	Intervention<br>(N=26)		Control<br>(N=28)		Total<br>(N=54)	
	N (%)	E	N (%)	E	N (%)	E
Embolic cerebral infarction	0 (0.00)	0	1 (3.57)	1	1 (1.85)	1
Epilepsy	1 (3.85)	1	0 (0.00)	0	1 (1.85)	1
Lacunar infarction	1 (3.85)	1	0 (0.00)	0	1 (1.85)	1
Myasthenia gravis	0 (0.00)	0	1 (3.57)	1	1 (1.85)	1
**Musculoskeletal and connective tissue disorders**	**5 (19.23)**	**7**	**5 (17.86)**	**7**	**10 (18.52)**	**14**
Osteoporosis	1 (3.85)	1	2 (7.14)	2	3 (5.56)	3
Rheumatoid arthritis	1 (3.85)	1	1 (3.57)	1	2 (3.70)	2
Spinal stenosis	0 (0.00)	0	2 (7.14)	2	2 (3.70)	2
Connective tissue disorder	1 (3.85)	1	0 (0.00)	0	1 (1.85)	1
Osteoarthritis	1 (3.85)	1	0 (0.00)	0	1 (1.85)	1
Osteonecrosis	1 (3.85)	1	0 (0.00)	0	1 (1.85)	1
Osteopenia	1 (3.85)	1	0 (0.00)	0	1 (1.85)	1
Polymyalgia rheumatica	0 (0.00)	0	1 (3.57)	1	1 (1.85)	1
Rotator cuff syndrome	0 (0.00)	0	1 (3.57)	1	1 (1.85)	1
Systemic lupus erythematosus	1 (3.85)	1	0 (0.00)	0	1 (1.85)	1
**Hepatobiliary disorders**	**4 (15.38)**	**4**	**4 (14.29)**	**4**	**8 (14.81)**	**8**
Hepatic cirrhosis	1 (3.85)	1	2 (7.14)	2	3 (5.56)	3
Autoimmune hepatitis	0 (0.00)	0	1 (3.57)	1	1 (1.85)	1
Cholecystitis acute	0 (0.00)	0	1 (3.57)	1	1 (1.85)	1
Chronic hepatitis	1 (3.85)	1	0 (0.00)	0	1 (1.85)	1
Hepatic steatosis	1 (3.85)	1	0 (0.00)	0	1 (1.85)	1
Liver disorder	1 (3.85)	1	0 (0.00)	0	1 (1.85)	1
**Renal and urinary disorders**	**4 (15.38)**	**4**	**3 (10.71)**	**4**	**7 (12.96)**	**8**
Acute kidney injury	1 (3.85)	1	2 (7.14)	2	3 (5.56)	3
Chronic kidney disease	1 (3.85)	1	1 (3.57)	1	2 (3.70)	2
Bladder dysfunction	0 (0.00)	0	1 (3.57)	1	1 (1.85)	1

System Organ Class<br>Preferred Terms	Intervention (N=26)		Control (N=28)		Total (N=54)	
	N (%)	E	N (%)	E	N (%)	E
Dysuria	1 (3.85)	1	0 (0.00)	0	1 (1.85)	1
Hydronephrosis	1 (3.85)	1	0 (0.00)	0	1 (1.85)	1
**Blood and lymphatic system disorders**	**2 (7.69)**	**2**	**4 (14.29)**	**4**	**6 (11.11)**	**6**
Anemia	1 (3.85)	1	3 (10.71)	3	4 (7.41)	4
Aplastic anemia	0 (0.00)	0	1 (3.57)	1	1 (1.85)	1
Thrombocytopenia	1 (3.85)	1	0 (0.00)	0	1 (1.85)	1
**Psychiatric disorders**	**3 (11.54)**	**3**	**2 (7.14)**	**2**	**5 (9.26)**	**5**
Depression	2 (7.69)	2	1 (3.57)	1	3 (5.56)	3
Depressed mood	1 (3.85)	1	0 (0.00)	0	1 (1.85)	1
Insomnia	0 (0.00)	0	1 (3.57)	1	1 (1.85)	1
**Gastrointestinal disorders**	**3 (11.54)**	**3**	**0 (0.00)**	**0**	**3 (5.56)**	**3**
Gastroesophageal reflux disease	2 (7.69)	2	0 (0.00)	0	2 (3.70)	2
Superior mesenteric artery dissection	1 (3.85)	1	0 (0.00)	0	1 (1.85)	1
**Injury, poisoning, and procedural complications**	**1 (3.85)**	**1**	**2 (7.14)**	**2**	**3 (5.56)**	**3**
Radiation pneumonitis	0 (0.00)	0	1 (3.57)	1	1 (1.85)	1
Spinal cord injury	1 (3.85)	1	0 (0.00)	0	1 (1.85)	1
Thermal burn	0 (0.00)	0	1 (3.57)	1	1 (1.85)	1
**Reproductive system and breast disorders**	**1 (3.85)**	**1**	**2 (7.14)**	**2**	**3 (5.56)**	**3**
Benign prostatic hyperplasia	1 (3.85)	1	2 (7.14)	2	3 (5.56)	3
**Endocrine disorders**	**1 (3.85)**	**1**	**1 (3.57)**	**1**	**2 (3.70)**	**2**
Hypothyroidism	1 (3.85)	1	1 (3.57)	1	2 (3.70)	2
**Skin and subcutaneous tissue disorders**	**1 (3.85)**	**1**	**1 (3.57)**	**1**	**2 (3.70)**	**2**
Dermatomyositis	1 (3.85)	1	0 (0.00)	0	1 (1.85)	1
Skin necrosis	0 (0.00)	0	1 (3.57)	1	1 (1.85)	1
**Surgical and medical procedures**	**1 (3.85)**	**1**	**1 (3.57)**	**1**	**2 (3.70)**	**2**
Cord blood transplant therapy	0 (0.00)	0	1 (3.57)	1	1 (1.85)	1

System Organ Class<br>Preferred Terms	Intervention (N=26)		Control (N=28)		Total (N=54)	
	**N (%)**	**E**	**N (%)**	**E**	**N (%)**	**E**
Thoracic cavity drainage	1 (3.85)	1	0 (0.00)	0	1 (1.85)	1
**Ear and labyrinth disorders**	**1 (3.85)**	**1**	**0 (0.00)**	**0**	**1 (1.85)**	**1**
Hypoacusis	1 (3.85)	1	0 (0.00)	0	1 (1.85)	1
**Eye disorders**	**0 (0.00)**	**0**	**1 (3.57)**	**1**	**1 (1.85)**	**1**
Macular degeneration	0 (0.00)	0	1 (3.57)	1	1 (1.85)	1
**Immune system disorders**	**1 (3.85)**	**1**	**0 (0.00)**	**0**	**1 (1.85)**	**1**
Graft versus host disease	1 (3.85)	1	0 (0.00)	0	1 (1.85)	1

Note 1: Duplication permitted.

Note 2: Coding by System Organ Class (SOC) and Preferred Term (PT) of MedDRA (version 26.0)

-: Not analyzed

Table S3m. Baseline previous medication history of the participants in the FA set

Anatomical Therapeutic	Intervention (N=26)		Control (N=28)		Total (N=54)	
	N (%)	E	N (%)	E	N (%)	E
**Total**	**22 (84.62)**	**195**	**27 (96.43)**	**201**	**49 (90.74)**	**396**
p-value	0.1842&					
**Alimentary tract and metabolism**	**16 (61.54)**	**42**	**19 (67.86)**	**52**	**35 (64.81)**	**94**
Drugs for acid related disorders	7 (26.92)	10	12 (42.86)	13	19 (35.19)	23
Drugs used in diabetes	6 (23.08)	12	10 (35.71)	24	16 (29.63)	36
Antidiarrheals, intestinal anti-inflammatory/anti-infective agents	4 (15.38)	7	2 (7.14)	2	6 (11.11)	9
Bile and liver therapy	4 (15.38)	5	2 (7.14)	3	6 (11.11)	8
Antiemetics and antinauseants	2 (7.69)	2	1 (3.57)	1	3 (5.56)	3
Drugs for constipation	1 (3.85)	1	2 (7.14)	2	3 (5.56)	3
Drugs for functional gastrointestinal disorders	1 (3.85)	1	2 (7.14)	2	3 (5.56)	3
Mineral supplements	3 (11.54)	3	0 (0.00)	0	3 (5.56)	3
Vitamins	1 (3.85)	1	2 (7.14)	2	3 (5.56)	3
Other alimentary tract and metabolism products	0 (0.00)	0	2 (7.14)	3	2 (3.70)	3
**Cardiovascular system**	**15 (57.69)**	**41**	**16 (57.14)**	**39**	**31 (57.41)**	**80**
Lipid-modifying agents	6 (23.08)	7	11 (39.29)	14	17 (31.48)	21
Calcium channel blockers	6 (23.08)	6	10 (35.71)	11	16 (29.63)	17
Beta-blocking agents	8 (30.77)	8	5 (17.86)	6	13 (24.07)	14
Agents acting on the renin–angiotensin system	7 (26.92)	8	4 (14.29)	4	11 (20.37)	12
Cardiac therapy	5 (19.23)	8	1 (3.57)	1	6 (11.11)	9
Diuretics	3 (11.54)	3	0 (0.00)	0	3 (5.56)	3
Peripheral vasodilators	0 (0.00)	0	2 (7.14)	2	2 (3.70)	2
Antihypertensives	1 (3.85)	1	0 (0.00)	0	1 (1.85)	1
Vasoprotective agents	0 (0.00)	0	1 (3.57)	1	1 (1.85)	1

Anatomical Therapeutic	Intervention (N=26)		Control (N=28)		Total (N=54)	
	N (%)	E	N (%)	E	N (%)	E
**Blood and blood-forming organs**	**7 (26.92)**	**8**	**14 (50.00)**	**19**	**21 (38.89)**	**27**
Antithrombotic agents	5 (19.23)	5	10 (35.71)	13	15 (27.78)	18
Anti-anemic preparations	2 (7.69)	3	3 (10.71)	5	5 (9.26)	8
Blood substitutes and perfusion solutions	0 (0.00)	0	1 (3.57)	1	1 (1.85)	1
**Nervous system**	**8 (30.77)**	**26**	**12 (42.86)**	**22**	**20 (37.04)**	**48**
Psychoanaleptics	4 (15.38)	7	4 (14.29)	4	8 (14.81)	11
Other nervous system drugs	1 (3.85)	2	6 (21.43)	7	7 (12.96)	9
Psycholeptics	3 (11.54)	4	4 (14.29)	5	7 (12.96)	9
Analgesics	3 (11.54)	7	3 (10.71)	3	6 (11.11)	10
Anti-Parkinson's drugs	1 (3.85)	1	2 (7.14)	2	3 (5.56)	3
Antiepileptics	2 (7.69)	5	1 (3.57)	1	3 (5.56)	6
**Antineoplastic and immunomodulating agents**	**11 (42.31)**	**22**	**8 (28.57)**	**10**	**19 (35.19)**	**32**
Immunosuppressants	6 (23.08)	9	4 (14.29)	4	10 (18.52)	13
Antineoplastic agents	4 (15.38)	9	3 (10.71)	4	7 (12.96)	13
Endocrine therapy	3 (11.54)	3	2 (7.14)	2	5 (9.26)	5
Immunostimulants	1 (3.85)	1	0 (0.00)	0	1 (1.85)	1
**Systemic hormonal preparations, excluding sex hormones and insulins**	**11 (42.31)**	**15**	**8 (28.57)**	**9**	**19 (35.19)**	**24**
Corticosteroids for systemic use	9 (34.62)	13	6 (21.43)	6	15 (27.78)	19
Thyroid therapy	2 (7.69)	2	2 (7.14)	2	4 (7.41)	4
Calcium homeostasis	0 (0.00)	0	1 (3.57)	1	1 (1.85)	1
**Anti-infectives for systemic use**	**9 (34.62)**	**15**	**8 (28.57)**	**13**	**17 (31.48)**	**28**
Antibacterial agents for systemic use	5 (19.23)	9	4 (14.29)	4	9 (16.67)	13
Antiviral agents for systemic use	3 (11.54)	3	4 (14.29)	5	7 (12.96)	8
Antimycotic agents for systemic use	0 (0.00)	0	3 (10.71)	3	3 (5.56)	3
Antimycobacterial agents	1 (3.85)	2	1 (3.57)	1	2 (3.70)	3

Anatomical Therapeutic	Intervention (N=26)		Control (N=28)		Total (N=54)	
	N (%)	E	N (%)	E	N (%)	E
Immune sera and immunoglobulins	1 (3.85)	1	0 (0.00)	0	1 (1.85)	1
**Respiratory system**	**9 (34.62)**	**15**	**8 (28.57)**	**13**	**17 (31.48)**	**28**
Cough and cold preparations	3 (11.54)	5	6 (21.43)	6	9 (16.67)	11
Drugs for obstructive airway diseases	3 (11.54)	4	4 (14.29)	6	7 (12.96)	10
Antihistamines for systemic use	3 (11.54)	3	0 (0.00)	0	3 (5.56)	3
Nasal preparations	3 (11.54)	3	0 (0.00)	0	3 (5.56)	3
Other respiratory system products	0 (0.00)	0	1 (3.57)	1	1 (1.85)	1
**Genitourinary system and sex hormones**	**3 (11.54)**	**3**	**6 (21.43)**	**11**	**9 (16.67)**	**14**
Urological agents	3 (11.54)	3	6 (21.43)	11	9 (16.67)	14
**Musculoskeletal system**	**2 (7.69)**	**3**	**5 (17.86)**	**7**	**7 (12.96)**	**10**
Anti-inflammatory and antirheumatic products	1 (3.85)	1	4 (14.29)	5	5 (9.26)	6
Muscle relaxants	1 (3.85)	2	1 (3.57)	1	2 (3.70)	3
Drugs for treatment of bone diseases	0 (0.00)	0	1 (3.57)	1	1 (1.85)	1
**Antiparasitic products, insecticides, and repellents**	**2 (7.69)**	**2**	**2 (7.14)**	**2**	**4 (7.41)**	**4**
Antiprotozoals	2 (7.69)	2	2 (7.14)	2	4 (7.41)	4
**Various/others**	**2 (7.69)**	**2**	**1 (3.57)**	**1**	**3 (5.56)**	**3**
General nutrients	1 (3.85)	1	1 (3.57)	1	2 (3.70)	2
All other therapeutic products	1 (3.85)	1	0 (0.00)	0	1 (1.85)	1
**Dermatological agents**	**1 (3.85)**	**1**	**1 (3.57)**	**1**	**2 (3.70)**	**2**
Antibiotics and chemotherapeutics for dermatological use	1 (3.85)	1	1 (3.57)	1	2 (3.70)	2
**Sensory organs**	**0 (0.00)**	**0**	**1 (3.57)**	**2**	**1 (1.85)**	**2**
Ophthalmological agents	0 (0.00)	0	1 (3.57)	2	1 (1.85)	2

Note 1: Duplication permitted.

Note 2: Coding by the Anatomical Therapeutic Chemical (ATC) classification system (version 2023) of the

World Health Organization

&: Fisher's exact test

Table S3n. Baseline concomitant medications of the FA participants in the set

Anatomical Therapeutic	Intervention (N=26)		Control (N=28)		Total (N=54)	
	N (%)	E	N (%)	E	N (%)	E
**Total**	**16 (61.54)**	**76**	**15 (53.57)**	**78**	**31 (57.41)**	**154**
p-value	0.5541$					
**Cardiovascular system**	**8 (30.77)**	**18**	**6 (21.43)**	**13**	**14 (25.93)**	**31**
Lipid-modifying agents	4 (15.38)	5	5 (17.86)	5	9 (16.67)	10
Beta-blocking agents	3 (11.54)	3	2 (7.14)	3	5 (9.26)	6
Agents acting on the renin–angiotensin system	3 (11.54)	3	1 (3.57)	1	4 (7.41)	4
Calcium channel blockers	3 (11.54)	3	1 (3.57)	2	4 (7.41)	5
Cardiac therapy	2 (7.69)	3	0 (0.00)	0	2 (3.70)	3
Diuretics	1 (3.85)	1	0 (0.00)	0	1 (1.85)	1
Peripheral vasodilators	0 (0.00)	0	1 (3.57)	1	1 (1.85)	1
Vasoprotective agents	0 (0.00)	0	1 (3.57)	1	1 (1.85)	1
**Alimentary tract and metabolism**	**4 (15.38)**	**15**	**7 (25.00)**	**16**	**11 (20.37)**	**31**
Drugs used in diabetes	2 (7.69)	4	3 (10.71)	6	5 (9.26)	10
Drugs for acid related disorders	1 (3.85)	3	3 (10.71)	3	4 (7.41)	6
Bile and liver therapy	2 (7.69)	3	1 (3.57)	1	3 (5.56)	4
Antidiarrheals, intestinal anti-inflammatory/anti-infective agents	1 (3.85)	2	1 (3.57)	1	2 (3.70)	3
Drugs for constipation	1 (3.85)	2	1 (3.57)	1	2 (3.70)	3
Vitamins	1 (3.85)	1	1 (3.57)	1	2 (3.70)	2
Drugs for functional gastrointestinal disorders	0 (0.00)	0	1 (3.57)	1	1 (1.85)	1

Anatomical Therapeutic	Intervention (N=26)		Control (N=28)		Total (N=54)	
	N (%)	E	N (%)	E	N (%)	E
Other alimentary tract and metabolism products	0 (0.00)	0	1 (3.57)	2	1 (1.85)	2
**Nervous system**	**6 (23.08)**	**16**	**5 (17.86)**	**12**	**11 (20.37)**	**28**
Psychoanaleptics	3 (11.54)	6	2 (7.14)	2	5 (9.26)	8
Psycholeptics	3 (11.54)	4	2 (7.14)	2	5 (9.26)	6
Other nervous system drugs	1 (3.85)	1	3 (10.71)	4	4 (7.41)	5
Analgesics	2 (7.69)	4	1 (3.57)	1	3 (5.56)	5
Anti-Parkinson drugs	1 (3.85)	1	2 (7.14)	2	3 (5.56)	3
Antiepileptics	0 (0.00)	0	1 (3.57)	1	1 (1.85)	1
**Antineoplastic and immunomodulating agents**	**5 (19.23)**	**5**	**5 (17.86)**	**6**	**10 (18.52)**	**11**
Immunosuppressants	2 (7.69)	2	3 (10.71)	3	5 (9.26)	5
Endocrine therapy	2 (7.69)	2	1 (3.57)	1	3 (5.56)	3
Antineoplastic agents	1 (3.85)	1	1 (3.57)	2	2 (3.70)	3
**Systemic hormonal preparations, excluding sex hormones and insulins**	**4 (15.38)**	**5**	**6 (21.43)**	**6**	**10 (18.52)**	**11**
Corticosteroids for systemic use	3 (11.54)	3	4 (14.29)	4	7 (12.96)	7
Thyroid therapy	2 (7.69)	2	2 (7.14)	2	4 (7.41)	4
**Blood and blood-forming organs**	**3 (11.54)**	**3**	**6 (21.43)**	**7**	**9 (16.67)**	**10**
Antithrombotic agents	2 (7.69)	2	4 (14.29)	5	6 (11.11)	7
Anti-anemic preparations	1 (3.85)	1	1 (3.57)	1	2 (3.70)	2
Blood substitutes and perfusion solutions	0 (0.00)	0	1 (3.57)	1	1 (1.85)	1
**Anti-infectives for systemic use**	**5 (19.23)**	**8**	**2 (7.14)**	**3**	**7 (12.96)**	**11**
Antibacterial agents for systemic use	3 (11.54)	5	0 (0.00)	0	3 (5.56)	5
Antimycobacterial agents	1 (3.85)	1	1 (3.57)	1	2 (3.70)	2
Antivirals for systemic use	1 (3.85)	1	1 (3.57)	1	2 (3.70)	2
Antimycotics for systemic use	0 (0.00)	0	1 (3.57)	1	1 (1.85)	1

Anatomical Therapeutic	Intervention (N=26)		Control (N=28)		Total (N=54)	
	N (%)	E	N (%)	E	N (%)	E
Immune sera and immunoglobulins	1 (3.85)	1	0 (0.00)	0	1 (1.85)	1
**Respiratory system**	**3 (11.54)**	**3**	**3 (10.71)**	**5**	**6 (11.11)**	**8**
Drugs for obstructive airway diseases	0 (0.00)	0	3 (10.71)	4	3 (5.56)	4
Cough and cold preparations	1 (3.85)	1	1 (3.57)	1	2 (3.70)	2
Antihistamines for systemic use	1 (3.85)	1	0 (0.00)	0	1 (1.85)	1
Nasal preparations	1 (3.85)	1	0 (0.00)	0	1 (1.85)	1
**Genitourinary system and sex hormones**	**2 (7.69)**	**2**	**2 (7.14)**	**4**	**4 (7.41)**	**6**
Urological agents	2 (7.69)	2	2 (7.14)	4	4 (7.41)	6
**Musculoskeletal system**	**1 (3.85)**	**1**	**2 (7.14)**	**2**	**3 (5.56)**	**3**
Anti-inflammatory and antirheumatic products	1 (3.85)	1	1 (3.57)	1	2 (3.70)	2
Muscle relaxants	0 (0.00)	0	1 (3.57)	1	1 (1.85)	1
**Dermatological agents**	**0 (0.00)**	**0**	**2 (7.14)**	**2**	**2 (3.70)**	**2**
Antibiotics and chemotherapeutics for dermatological use	0 (0.00)	0	1 (3.57)	1	1 (1.85)	1
Corticosteroids, dermatological preparations	0 (0.00)	0	1 (3.57)	1	1 (1.85)	1
**Antiparasitic products, insecticides, and repellents**	**0 (0.00)**	**0**	**1 (3.57)**	**1**	**1 (1.85)**	**1**
Antiprotozoal agents	0 (0.00)	0	1 (3.57)	1	1 (1.85)	1
**Various**	**0 (0.00)**	**0**	**1 (3.57)**	**1**	**1 (1.85)**	**1**
General nutrients	0 (0.00)	0	1 (3.57)	1	1 (1.85)	1

Note 1: Duplication permitted. Note 2: Coding by the Anatomical Therapeutic Chemical (ATC) classification system (version 2023) of the World Health Organization

$^{\$}$: Chi-square test

6. Efficacy outcome of participants in the FA set

Table S4a. The primary efficacy outcome in the FA set

	Intervention (N=26)	Control (N=28)	p-value
**Modified CPIS (score)**			
Change in score:			0.8656‡
(CPIS after 72 h − baseline CPIS)			
N	23	24	
Mean (SD)	-0.13 (1.58)	-0.58 (1.18)	
Median	0.00	-1.00	
Min, Max	-3.00, 4.00	-2.00, 2.00	
Intervention – control (97.5% confidence interval for difference)	0.45 (-∞, 1.27)		

Note 1: The following participants were excluded from the analysis because lack of CPIS after 72 hours: 3 patients in the intervention group (1S-001, 1S-018, and 1S-026) and 4 patients in the control group (1S-006, 1S-014, 1S-020, and 2S-007)

‡: Independent two-sample *t*-test

Table S4b. Secondary outcomes of participants in the FA set

	Intervention (N=26)	Control (N=28)	*p*-value
**Modified CPIS (score)**			
**At baseline**			0.7317#
N	26	28	
Mean (SD)	3.58 (1.84)	3.75 (1.84)	
Median	3.00	4.00	
Min, Max	1.00, 7.00	1.00, 7.00	
**After 72 ± 3 h**			0.7601#
N	23	24	
Mean (SD)	3.26 (1.48)	3.17 (1.58)	

	Intervention (N=26)	Control (N=28)	*p*-value
Median	3.00	3.50	
Min, Max	1.00, 6.00	1.00, 6.00	
**Change in the modified CPIS**			0.2941#
**(CPIS after 72 h – baseline CPIS)**			
N	23	24	
Mean (SD)	-0.13 (1.58)	-0.58 (1.18)	
Median	0.00	-1.00	
Min, Max	-3.00, 4.00	-2.00, 2.00	
p-value	0.7164§	0.0379§	
**Rate of change in the modified CPIS**			0.3195#
**(CPIS after 72 h − baseline CPIS)/baseline CPIS**			
N	23	24	
Mean (SD)	0.13 (0.70)	-0.03 (0.59)	
Median	0.00	-0.17	
Min, Max	-0.50, 2.00	-0.67, 2.00	
**Improvement rate in the modified CPIS, N (%)**			0.3852&
Improvement	9 (39.13)	13 (54.17)	
No improvement	14 (60.87)	11 (45.83)	

Note 1: Classified as "improvement" if the modified CPIS improved by 1 point or more after 72 ± 3 hours, and as "no improvement" if not

Note 2: The following participants were excluded from the analysis because of the lack of CPIS data after 72 hours: 3 patients in the intervention group (1S-001, 1S-018, and 1S-026) and 4 patients in the control group (1S-006, 1S-014, 1S-020, and 2S-007)

Note 3: The following participants showed "Improvement" in the modified CPIS: 9 patients in the intervention group (1S-003, 1S-008, 1S-010, 1S-016, 2S-006, 2S-008, 2S-009, 2S-015, and 2S-030) and 13 patients in the control group (1S-004, 1S-012, 1S-015, 1S-017, 1S-022, 1S-023, 2S-001, 2S-010, 2S-016, 2S-018, 2S-020, 2S-021, and 2S-028)

Note 4: The following participants showed "No improvement" in the modified CPIS: 14 patients in the

intervention group (1S-007, 1S-011, 1S-021, 1S-024, 2S-002, 2S-003, 2S-011, 2S-013, 2S-017, 2S-019, 2S-024, 2S-025, 2S-027, and 2S-031) and 11 patients in the control group (1S-002, 1S-005, 1S-009, 1S-019, 1S-025, 2S-004, 2S-005, 2S-012, 2S-014, 2S-023, and 2S-029)

#: Wilcoxon rank-sum test

§: Wilcoxon signed-rank test

&: Fisher's exact test

Table S4c. Number of suctions performed in addition to those specified in the protocol for 72 ± 3 hours in the FA set

	Intervention (N=26)	Control (N=28)	p-value
**Number of suctions performed in addition to those specified in the protocol**			0.3993#
N	26	28	
Mean (SD)	7.85 (6.66)	6.61 (6.21)	
Median	7.00	4.00	
Min, Max	0.00, 27.00	0.00, 22.00	

#: Wilcoxon rank-sum test

Table S4d. Total amount of secretions (cc) collected for 72 ± 3 h for participants in the FA set

	Intervention (N=26)	Control (N=28)	p-value
**Total secretion (cc)**			0.9432#
N	25	28	
Mean (SD)	153.84 (168.52)	134.25 (116.91)	
Median	86.00	140.50	
Min, Max	22.00, 660.00	0.00, 523.00	

Note 1: Total amount of secretions collected (cc) in the intervention group: total collected secretion (cc) – saline used during suction (cc) in the control group.

Note 2: One participant in intervention group (2S-011) was not included in the analysis because of the answer “No” to the question whether or not the amount of sputum was measured.

#: Wilcoxon rank-sum test

Table S4e. Results of the device satisfaction survey after 72 ± 3 h in the FA set

	Intervention (N=26)	Control (N=28)
**Device satisfaction survey (score)**		
N	23	
Mean (SD)	5.88 (1.05)	
Median	6.00	
Min, Max	4.13, 7.80	

Note 1: Analyzed only in the Intervention group

Note 2: The following participants were excluded from the analysis due to a lack of survey results: 3 patients in the intervention group (1S-001, 1S-018, and 1S-026)

S7. Safety outcome of participants in the safety set

Table S5a. Summary of adverse events that occurred during the study

Classification	Intervention (N=26)		Control (N=28)		Total (N=54)	
	N (%)	E	N (%)	E	N (%)	E
**Adverse events**	3 (11.54)	3	0 (0.00)	0	3 (5.56)	3
**Severe adverse events**	2 (7.69)	2	0 (0.00)	0	2 (3.70)	2
**Severity**						
Mild	1 (3.85)	1	0 (0.00)	0	1 (1.85)	1
Moderate	0 (0.00)	0	0 (0.00)	0	0 (0.00)	0
Severe	2 (7.69)	2	0 (0.00)	0	2 (3.70)	2
**Causal relation with medical device**						
Definitely related	0 (0.00)	0	0 (0.00)	0	0 (0.00)	0
Probably related	0 (0.00)	0	0 (0.00)	0	0 (0.00)	0
Possibly related	0 (0.00)	0	0 (0.00)	0	0 (0.00)	0
Possibly not related	0 (0.00)	0	0 (0.00)	0	0 (0.00)	0
Definitely not related	3 (11.54)	3	0 (0.00)	0	3 (5.56)	3
Unknown	0 (0.00)	0	0 (0.00)	0	0 (0.00)	0
**Action related to medical device**						
No action	1 (3.85)	1	0 (0.00)	0	1 (1.85)	1
Removal of device	2 (7.69)	2	0 (0.00)	0	2 (3.70)	2
**Treatment for adverse events**						
No treatment	3 (11.54)	3	0 (0.00)	0	3 (5.56)	3
Medication administration	0 (0.00)	0	0 (0.00)	0	0 (0.00)	0
Procedure or surgery	0 (0.00)	0	0 (0.00)	0	0 (0.00)	0
Other	0 (0.00)	0	0 (0.00)	0	0 (0.00)	0
**Result of adverse events**						
Recovery/No sequelae	1 (3.85)	1	0 (0.00)	0	1 (1.85)	1

Classification	Intervention (N=26)		Control (N=28)		Total (N=54)	
	N (%)	E	N (%)	E	N (%)	E
Recovery/With sequelae	0 (0.00)	0	0 (0.00)	0	0 (0.00)	0
In recovery period	0 (0.00)	0	0 (0.00)	0	0 (0.00)	0
Not recovered	0 (0.00)	0	0 (0.00)	0	0 (0.00)	0
Death	2 (7.69)	2	0 (0.00)	0	2 (3.70)	2
Unknown	(0.00)	0	0 (0.00)	0	0 (0.00)	0

Note 1: N (%), E: Number of cases

Table S5b. Adverse events classified by organ system

System Organ Class<br>Preferred Terms	Intervention (N=26)		Control (N=28)		Total (N=54)	
	N (%)	E	N (%)	E	N (%)	E
**Total**	**3 (11.54)**	**3**	**0 (0.00)**	**0**	**3 (5.56)**	**3**
p-value	0.1048&					
**Cardiac disorders**	**3 (11.54)**	**3**	**0 (0.00)**	**0**	**3 (5.56)**	**3**
Cardiac arrest	2 (7.69)	2	0 (0.00)	0	2 (3.70)	2
Atrial fibrillation	1 (3.85)	1	0 (0.00)	0	1 (1.85)	1

Note 1: N (%), E: Number of cases

Note 2: Coding by the System Organ Class (SOC) and Preferred Term (PT) of MedDRA (version 26.0)

&: Fisher's exact test

Table S5c. Tracheal mucosal injury in Safety set

	Intervention (N=26)	Control (N=28)	Total (N=54)	p-value
**Grade of tracheal mucosal injury, N (%)**				
At baseline				0.5796[&]
0	3 (13.04)	8 (33.33)	11 (23.40)	
1	9 (39.13)	7 (29.17)	16 (34.04)	
2	6 (26.09)	6 (25.00)	12 (25.53)	
3	3 (13.04)	2 (8.33)	5 (10.64)	
4	2 (8.70)	1 (4.17)	3 (6.38)	
After 72 ± 3 hours				0.2323[&]
0	8 (34.78)	8 (33.33)	16 (34.04)	
1	4 (17.39)	10 (41.67)	14 (29.79)	
2	6 (26.09)	5 (20.83)	11 (23.40)	
3	4 (17.39)	1 (4.17)	5 (10.64)	
4	1 (4.35)	0 (0.00)	1 (2.13)	
**Tracheal mucosal injury incidence rate (%), N (%)**	1 (3.85)	1 (3.57)	2 (3.70)	1.0000[&]

Note 1: Grade of tracheal mucosal injury N (%): participants (percentage); the percentage is calculated based on the number of participants included in each group

Note 2: Tracheal mucosal injury incidence rate, N (%): participants (percentage); the percentage is calculated based on the number of participants included in each group, which included 7 participants with missing data. (participants with missing data: 3 participants in the intervention group (1S-001, 1S-018, and 1S-026); 4 participants in the control group (1S-006, 1S-014, 1S-020, and 2S-007)

Note 3: The grade by bronchoscopy is as follows: 0: Normal trachea; 1: erythema or edema; 2: erosion; 3: hemorrhage; 4: ulceration of necrosis

Note 4: Tracheal mucosal injury incidence rate (%) was defined as increase in the grade of tracheal mucosal injury by more than 1 point at day 3 compared with that at day 0.

Note 5: Participants with incident tracheal mucosal injury: intervention group (1S-008), control group (2S-004)

&: Fisher's exact test

Figure S2. Improvement in tracheal mucosal injury in the PP set

A.

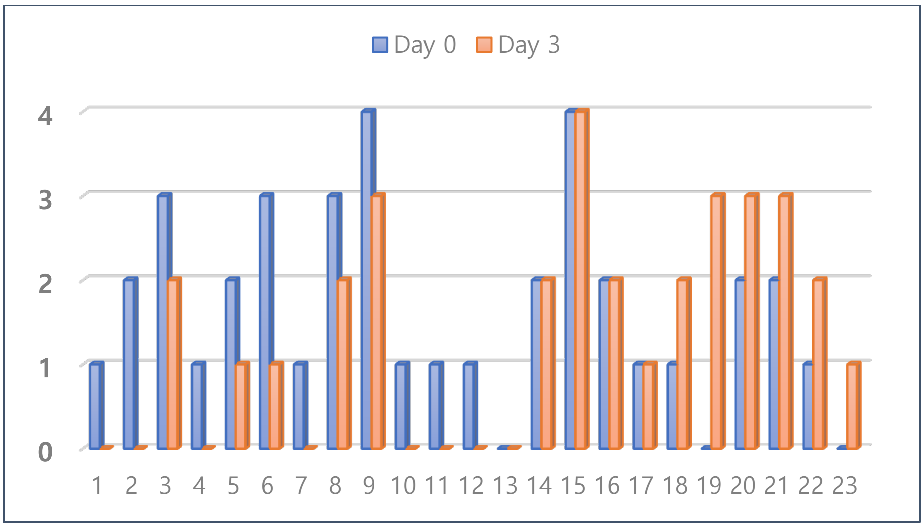

B.

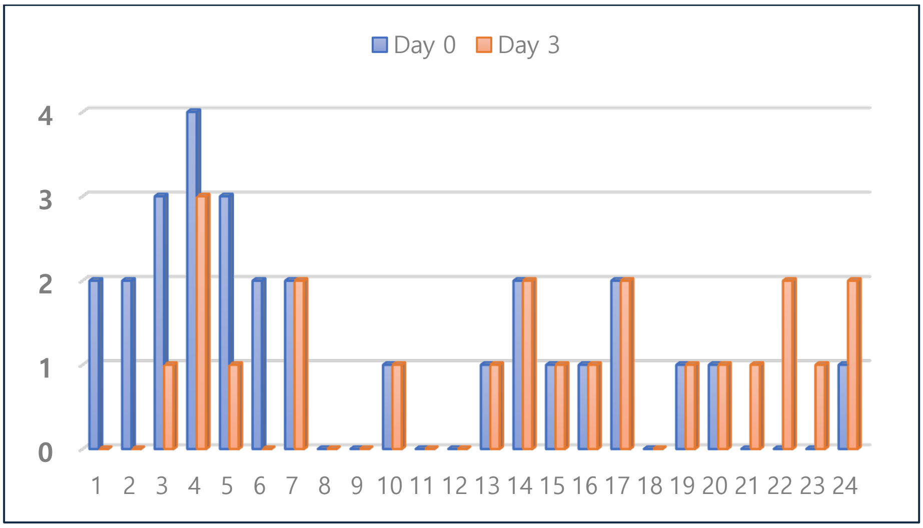

Supplementary Figure S2A shows the change in in the grade of tracheal mucosal injury in the intervention group. 12 participants (46.15%) in the intervention group showed improvement in tracheal mucosal injury. Supplementary Figure S2B shows the change in in the grade of tracheal mucosal injury in the control group. 6 participants (21.43%) in the control group showed improvement in the injury.

References

1. Cho, J.Y., et al: Pilot Study of Aerosolised Plus Intravenous Vancomycin in Mechanically Ventilated Patients with Methicillin-Resistant Staphylococcus Aureus Pneumonia. *J Clin Med* 2020;**9**(2).
2. Lee, H.W., et al: Clinical impact of early bronchoscopy in mechanically ventilated patients with aspiration pneumonia. *Respirology* 2015;**20**(7): p. 1115-22.
